# Dinos and GoPros: Children’s exploratory behaviors in a museum and their reflections on their learning

**DOI:** 10.3389/fpsyg.2023.1110612

**Published:** 2023-02-13

**Authors:** Deena Skolnick Weisberg, Lucretia C. Dunlap, David M. Sobel

**Affiliations:** ^1^Department of Psychological and Brain Sciences, Villanova University, Villanova, PA, United States; ^2^Department of Cognitive, Linguistic, and Psychological Sciences, Brown University, Providence, RI, United States

**Keywords:** museum, STEM learning, dinosaurs, exploration, informal education

## Abstract

Research in both laboratory and museum settings suggests that children’s exploration and caregiver–child interaction relate to children’s learning and engagement. Most of this work, however, takes a third-person perspective on children’s exploration of a single activity or exhibit, and does not consider children’s perspectives on their own exploration. In contrast, the current study recruited 6-to 10-year-olds (*N* = 52) to wear GoPro cameras, which recorded their first-person perspectives as they explored a dinosaur exhibition in a natural history museum. During a 10-min period, children were allowed to interact with 34 different exhibits, their caregivers and families, and museum staff however they wished. Following their exploration, children were asked to reflect on their exploration while watching the video they created and to report on whether they had learned anything. Children were rated as more engaged when they explored collaboratively with their caregivers. Children were more likely to report that they learned something when they were more engaged, and when they spent more time at exhibits that presented information didactically rather than being interactive. These results suggest that static exhibits have an important role to play in fostering learning experiences in museums, potentially because such exhibits allow for more caregiver–child interaction.

## Introduction

Over the last few decades, a growing body of work has focused on how exploratory behaviors, like play, serve as a foundation for learning (e.g., Rubin et al., 1983; Saracho, 1991; Pellegrini and Boyd, 1993; Lillard et al., 2013; Weisberg et al., 2016). Relations between exploration and learning have been studied both in the laboratory and in naturalistic environments, with studies on children’s naturalistic behaviors often seeking to translate findings on the relation between exploration and learning from the laboratory to more real-world settings (see, e.g., Callanan and Valle, 2008; Kline, 2015; Legare et al., 2017). To improve the ecological validity of studies of children’s learning from their exploration, researchers have begun to investigate the experiences that children and families have together in museum settings (e.g., Callanan, 2012; Sobel and Jipson, 2016). Museums offer children and families the opportunity to explore together, enabling researchers to study the interaction between children’s exploration and the dynamics of the family structure in a more authentic way (e.g., Allen, 2004; Gutwill and Allen, 2010; Falk and Dierking, 2018).

For example, many studies conducted in informal learning environments, such as children’s museums, examine the ways that children explore exhibits and how that exploration relates to their engagement with the exhibit content (e.g., Crowley et al., 2001; Fender and Crowley, 2007; Tare et al., 2011). Such studies also investigate what children might understand about their exploration through their reflections on their behaviors (e.g., Haden, 2010; Acosta et al., 2021; Marcus et al., 2021; see also McKeown and Gentilucci, 2007). However, most studies in museum settings focus on the ways in which children and their families interact at a particular exhibit or ask children to engage with a particular set of materials. Fewer studies consider how children explore large spaces within a museum, where many displays or exhibits compete for their attention. Because of this, it remains unclear how children’s exploration of larger museum spaces might relate to their engagement with the educational materials, to their beliefs about whether they learn from this material, and to the social interactions with caregivers and others who could serve to guide their learning.

Further, most of the prior work on children’s exploration and learning in museum settings have examined children’s behaviors and interactions from a third-person perspective, and children have only been asked to reflect on their experiences from memory. To begin to address these issues, the current study examined children’s exploration of a two-story dinosaur exhibition in a natural history museum (the Academy of Natural Sciences in Philadelphia). Six-to 10-year-olds wore head-mounted GoPro cameras to record their first-person perspective as they explored the exhibition. During a 10-minute period, children were allowed to interact with different exhibits, their caregivers and families, and museum staff however they wished. This way of capturing children’s experiences within a museum setting provides us with a unique view of how children explore scientific information while interacting with caregivers and others.

## Children’s exploration in museum settings

While there are several datasets that aim to capture naturalistic first-person data from babies and children (e.g., Smith et al., 2011; Jung et al., 2018; Slone et al., 2018; Sullivan et al., 2021), our design was inspired by a study conducted at Providence Children’s Museum, which also used GoPro cameras to capture children’s first-person perspectives on their interactions with the exhibit elements (Sobel et al., 2022b). That study focused on how children set goals for these interactions and the circumstances under which they changed those goals or their approach to achieving those goals. One of the main findings from that study was that children tended to be more engaged by the exhibits and to stay longer when they set their own goals, rather than when they followed goals suggested by the museum. In addition, children were more likely to revise their behaviors to try to achieve their goals when they interacted collaboratively with others (usually parents), as opposed to when they were acting alone or in parallel with others.

While that study conducted a similar investigation to the one reported here, Providence Children’s Museum differs in several key ways from the current museum context, allowing the current work to make novel contributions to our understanding of how children explore in museum spaces. One of the primary differences is that the Providence Children’s Museum exhibition was almost entirely interactive; children engaged with hands-on activities designed to prompt spatial thinking, such as a SOMA cube or Jovo blocks. The dinosaur exhibition investigated here had a few interactive elements but was primarily designed around having visitors view artifacts and read about them on informational plaques. This fundamentally changes the type of interactions that children are able to have with the exhibition. Indeed, the fact that the dinosaur exhibition included both interactive and didactic (or static) elements allowed us to investigate how these different kinds of exhibits affected children’s engagement and their interactions with their caregivers. While much work in developmental psychology suggests that children learn effectively from hands-on experiences (e.g., Schulz et al., 2007; Chi, 2009; Lapidow and Walker, 2020; Nussenbaum et al., 2020; Sobel et al., 2022a), static exhibits have different strengths and can also inspire children’s engagement in museums (e.g., Peart and Kool, 1988; Tunnicliffe and Scheersoi, 2009; Dancstep et al., 2015). One important question for the current study is thus the impact that these different kinds of experiences can have.

Second, the spatial thinking exhibition at Providence Children’s Museum was only about 1,000 square feet in total area, and was stanchioned off. Parents could sit at one end of the space while their children played, knowing that their children were safely confined. As a result, only 20% of children’s recorded play in that study was with their parent. In contrast, the dinosaur exhibition at the Academy of Natural Sciences was about 10,000 square feet in area and was spread across two floors. Caregivers thus often stayed nearby their children at all times, given the size of the museum and the number of visitors.

Another key difference is that the exhibit at Providence Children’s Museum, while focused on encouraging children’s spatial thinking, did not aim to teach particular pieces of scientific information. By contrast, the dinosaur exhibition did have this goal, aiming to teach visitors about different kinds of dinosaurs, ways in which dinosaurs are similar to and different from currently living species, and the scientific process of paleontology. Additionally, the dinosaur exhibition at the Academy of Natural Sciences was geared toward a much wider range of ages than the spatial thinking exhibit at Providence Children’s Museum. Practically speaking, this meant that many of the exhibits presented text and other didactic elements that required adults to interpret them for younger visitors. These differences necessitated different approaches to data coding and analysis in the current study. In particular, the current study aimed to investigate how children’s interactions with the exhibit elements and with their caregivers might shape their experiences of the exhibition.

Although we did not study play behavior directly, this focus draws theoretically on the framework of *guided play*, which involves a tradeoff between adult scaffolding and child autonomy, and which is beneficial to achieving learning goals (Weisberg et al., 2013, 2016). As in studies of guided play, the current work aimed to shed light on how adult-child interaction can help or hinder children’s learning and engagement. Previous museum-based studies have followed this framework and have similarly focused on the relation between parent–child interaction during children’s exploration of STEM-based exhibits and their learning. To take one example, Sobel et al. (2021) asked 4-to 7-year-olds and their parents to play together at a circuit exhibit. They coded the ways in which parents and children interacted in terms of *goal setting* – who set goals for the ways in which the dyad played. Some dyads were more parent directed, in which parents set goals for the play. Others were child directed, in which parents were more hands-off and allowed children to set goals. Still others were jointly directed, in which goals were set collaboratively, or parents were more supportive of how their children set goals. This study found that parental goal setting directly related to how engaged children were with a set of circuit construction challenges that were presented to children on their own (see also Fung and Callanan, 2013; Callanan et al., 2020; Medina and Sobel, 2020). In light of this, another goal of the present study is to confirm these results in the larger, more open setting of the dinosaur exhibition, investigating whether the relation between adult goal setting and children’s engagement extends to exploration across a set of exhibits, as opposed to their engagement with a single activity.

To that end, in addition to looking at the ways that adults might set goals for their children’s exploration of the space, we also considered one facet of the interaction between caregivers and children in more detail, which we called *juicy moments*. This aspect of our investigation was inspired by work by Gutwill and Allen (2010), which showed that encouraging families to develop ‘juicy questions’ about exhibits – questions that can be answered by interacting with the exhibit – families were more likely to set goals and generate explanations related to the questions. The families in that study also spent more time at the exhibits, suggesting children were more engaged by the experience. The current study did not explicitly ask families to generate such questions; rather, the point of connection between our investigation and theirs is in considering how ‘juicy’ aspects of a museum visit (instantiated here a moments of particularly rich engagement or of potential learning) relate to the nature of the exhibit or to how children reflect on their experience.

## Children’s reflections on their exploration

How children talk about their exploration in museums reflects what they understand about their experiences and their later learning (e.g., Haden, 2010). For instance, Marcus et al. (2017) showed that when children were presented with causal information during parent–child interaction in a museum, the children talked more about that causal knowledge when they reflected on their experience, even 2 weeks after their visit. Such causal knowledge also transfers to challenges presented in the home a week after their visit (Marcus et al., 2021). Similarly, the more STEM-based talk parents generated while playing with their children at STEM-related exhibits, the more STEM-related content children generated when asked to reflect on the activity (Acosta et al., 2021). These data suggest that reflection is an important component of children’s memory for and understanding of an exhibit and potentially what they learn from exploratory contexts like play.

What is not studied as much is the extent to which children make metacognitive judgments about whether they learned from their experience at the museum. Several laboratory-based studies suggest that children undergo significant development regarding the metacognitive capacity to reflect on their own learning between the ages of approximately 5 and 8 (Esbensen et al., 1997; Bartsch et al., 2003; Bemis et al., 2011, 2013; Tang and Bartsch, 2012; Sobel and Letourneau, 2015). Moreover, during this same age range, children also begin to appreciate the distinctions between learning and play and the relations between them, such as the idea that learning can occur through play (e.g., Howard et al., 2006; Letourneau and Sobel, 2020). In order to capture how children conceptualized their own learning in this exhibition, we showed children the GoPro video that they recorded of their own exploration and asked them to reflect on why they went to a particular exhibit, what they were doing and thinking about while at that exhibit, and whether they learned from their exploration (as in our prior study at the Providence Children’s Museum; Sobel et al., 2022b). Our goal with these questions was to document how children reflected on their own experiences of exploration and learning, whether they believed they had learned anything from the exploration, and, if so, whether there was any aspect of their exploratory behavior that predicted their saying that they had learned something.

Finally, as noted above, one important reason to conduct museum-based investigations is to gain insight into children’s behavior and social interactions in naturalistic contexts, breaking down the barrier between the laboratory and the real world. But an important difference between museum-based and lab-based studies on children’s exploratory behavior is that the museum-based work reported here does not include a direct measure of learning, only children’s reports of whether they thought they had learned something and the ways in which they talked about their experiences at the exhibits. Although the dinosaur exhibition that we investigated was designed to be pedagogical, different aspects of the exhibition aimed to teach different pieces of information. Because children were allowed to explore freely, not every child visited the same set of exhibits, which did not allow us to construct a measure of children’s learning that would be consistent across participants. More importantly, children entered the exhibition with different amounts of knowledge about dinosaurs and paleontology; some of our participants had even visited this exhibition before. A pre-test of children’s knowledge could allow us to equate for those differences, but asking children specific questions before their exploration would likely have skewed their attention to different aspects of the exhibition and changed how they explored, damaging our ability to observe truly naturalistic behavior. For those reasons, the main goal of the current investigation was not to measure what children learned *per se*, but rather their beliefs about whether they learned.

## The current study

Children’s engagement and learning are affected by the way in which their caregivers interact with them, particularly the extent to which caregivers let their children set goals autonomously. One major goal of the current investigation is to explore those effects in a more naturalistic set of interactions in order to clarify how these kinds of interactions can lead to beneficial outcomes. In turn, the results of this project can suggest ways to encourage these kinds of interactions in informal learning environments.

A second major goal of the current investigation is to probe more deeply how children conceptualize their own learning in a museum setting and how they reflect on their own actions during their exploration of the museum. Most of the prior work on children’s scientific thinking and causal reasoning in early childhood tends to focus on children’s first-order learning about novel causal systems, and does not consider children’s metacognitive views of their own learning. Despite this, metacognitive reflection plays a vital role in the development of children’s scientific thinking (Kuhn, 2007; Weisberg and Sobel, 2022). This project begins to explore these questions in two ways: (1) by capturing moments in children’s exploration where they seemed to be having particularly rich and important experiences (juicy moments), and (2) through a post-exploration interview, in which participants were shown key moments from the video that they created on their head-mounted GoPro during their exploration and were asked to reflect on what they were doing and why.

This combination of children’s first-person perspectives during their exploration of the exhibition and their post-exploration reflections allows us to probe in detail what sparks children’s engagement with museum exhibits as well as what insights they might have about their own exploratory behaviors. Although the rich data set that we have collected here can allow for many different investigations into different aspects of children’s experiences, the current study focuses primarily on correlates of children’s engagement and on relations between their engagement and their own reports of their exploratory behaviors. These analyses can provide unique insights into the basis of science learning in museums and other informal settings.

Finally, the nature of this exhibition allowed for another facet of considering caregiver–child interaction and its relation to children’s learning and engagement: the specific design of the exhibits. Some of the exhibits were static, designed primarily to be examined visually and presenting textual material to read. These exhibits didactically communicated explicit pieces of information about dinosaurs and paleontology. Other exhibits were more interactive, affording hands-on experiences and actions on the part of children and other visitors that might produce learning. Comparison of these types of exhibits, and their relation to children’s reflections and what children say about whether they learned, is of interest to thinking about the pedagogy of how information is presented in museum settings.

## Methods

### Participants

We recruited all the participants in this study while they were inside a dinosaur exhibition of a local natural history museum. The final sample includes 52 focal children between the ages of 6 and 10 years (mean age in months = 96.08,^1^ SD = 14.49), who participated together with whoever they had come to the museum with (caregivers, siblings, etc.). Three additional children were consented, but either chose not to participate in the study (*n* = 1) or ended their exploration after only a few minutes (*n* = 2). Data collection occurred between September 2019 and February 2020. We had planned to collect data from 60 children to match the sample size in Sobel et al. (2022b), but data collection was interrupted by the COVID-19 pandemic. We were unable to complete the sample when the museum reopened because the space had been reorganized to accommodate distancing requirements, so any additional observations would not have been adequately comparable to the original sample.

Our sample included 20 female and 32 male children. Of the 49 participants whose parents or guardians reported their race, there were 39 white participants, 6 Black participants, and 4 mixed-race participants. Additionally, 7 participants identified as Hispanic or Latino and 10 identified as not Hispanic or Latino; the remaining participants did not respond to this question. Parents also were asked to fill out a questionnaire reporting on other demographic factors and their views about science; more information about responses to this questionnaire can be found in the Supplementary materials.

For each participant, we identified the caregiver with whom they interacted the most in order to analyze caregiver–child interactions. Of these caregivers, 28 were female and 24 were male. Again, more information about the demographics of the sample are presented in Supplementary materials.

### Exhibition

This study focused on children’s exploration of the Dinosaur Hall exhibition at the Academy of Natural Sciences in Philadelphia. This exhibition stretches over two floors just to the right of the main entrance to the museum and is often the first place that families come after visiting the admissions desk.

For the purposes of our analyses, in consultation with curators and other museum staff, we divided the exhibition up into 34 exhibits (one participant experienced an additional special exhibit involving live chickens that was only available for that participant). Seven of the exhibits were classified as interactive, and the other 27 were classified as static. Interactive exhibits allow visitors to engage in actions that have an effect on the exhibit, such as the “Big Dig,” where visitors can brush away shredded cork pieces to find replica dinosaur bones, and a treadmill that is connected to a dinosaur skeleton, so that visitors who walk on the treadmill can make the skeleton move. Static exhibits present fossils, bones, or other artifacts, and visitors can read information about dinosaurs or paleontologists from plaques. See Supplementary Table S1 for a description of all of the exhibits and their classification as static or interactive. A map of the space with thumbnail photographs of the 34 exhibits can be found on OSF.^2^

### Procedure

#### Exploration

In this study, participants wore a head-mounted GoPro camera to record their first-person perspective as they explored the dinosaur exhibition. Children were allowed to interact however they wished with different exhibits, their caregivers and families, and museum staff. A research assistant followed each participant with a second GoPro camera (chest-mounted), recording a third-person perspective on what the participant was doing. During data collection sessions, we posted signage at the entrances to the exhibition informing museum visitors that we would be video-recording in this exhibition for research purposes, so they could choose to avoid the exhibition if they did not want to be recorded.

Participants were given 10 minutes to explore freely before proceeding to the post-exploration interview (see below). The research assistant gave the child a warning at 8 minutes that their time was almost over. We chose to end the exploration period after 10 minutes partially to match the method used in a previous study of children’s museum exploration using GoPros (Sobel et al., 2022b), but also to impose some experimental control for the sake of the reflection interviews; we wanted all children to have the same amount of time exploring to reflect on in the interviews. Moreover, limiting the time spent exploring ensured that the length of the overall research session was roughly the same for all participants, thereby not affecting their experience visiting the museum differently.

All videos that parents provided permission to share are available on Databrary.^3^

#### Post-exploration interview

Following the exploration period, children engaged in an interview that was similar the one used in prior work (Sobel et al., 2022b).

The first thing that happened in this interview was that the research assistant who had followed the child during the exploration period asked them to reflect on their exploration. To do so, the research assistant used the GoPro app on an iPad to pull up the first-person footage that the participant had recorded during their exploration. She scrubbed through this footage to find key moments in the participant’s visit, using the video as a reminder to the participant of the exhibits that they had visited. For each of these moments, the research assistant asked participants (1) why they chose to go to that exhibit, (2) what they were doing there, and (3) what they were thinking about. To keep the post-exploration interviews brief, participants were not asked about every exhibit that they had visited. Instead, the research assistant always asked about the first exhibit that the participant visited and then chose a few other exhibits that the research assistant judged to have included particularly interesting interactions or particularly meaningful engagement (following the same procedure described in Sobel et al., 2022b).

At the end of these reflections, the research assistant asked whether they had learned anything during their museum exploration. If the participant responded that they had, they were asked what they learned and how they learned it. If the participant responded that they had not, they were asked what they were doing and whether they could have been learning while they were engaged in whatever other activity that they named.

Children were also asked a set of questions regarding their understanding of learning (following work by Sobel and Letourneau, 2015). The results of this interview were unrelated to the analyses reported here, and these data are reported in the Supplemental materials section.

The full script for the post-exploration interview can be found on OSF.^4^

### Coding

#### Visit metrics

Children were coded as having visited a particular exhibit if they were physically present at it or looking at it for at least 5 seconds. All exploration videos were transcribed and participant behaviors (e.g., pointing) were coded using Datavyu; these coding files are available together with participants’ videos on Databrary (see footnote 3).

#### Child engagement

We coded how engaged each child was during the exploration period on a scale of 1 to 5, with 1 indicating no engagement and 5 indicating high engagement. Each child received a single code reflecting their overall level of engagement. Behaviors indicative of higher engagement involved the child showing clear enthusiasm for or interest in the exhibits, for example, asking questions, actively reading placards, touching or interacting with exhibits, and so on. Coders were thus instructed to pay attention to facial expressions, body language, verbal content, and the variety of exhibits that the child visited. Importantly, because children received a single score for engagement for the entire exploration period, this score did not simply reflect the amount of time spent at any particular exhibit. Rather, it aimed to holistically capture children’s behavior across the entire exploration period. A team of two coders, one of whom was the second author, independently coded each video in the set. The coders met after every 5 videos to discuss and reconcile any discrepancies. Cronbach’s alpha for agreement between the two coders was 0.97.

#### Caregiver–child interaction

A separate set of three coders, together with the second author, identified a primary caregiver for each participant and coded the child’s interactions with this caregiver, again on a scale of 1 to 5. Following prior work on caregiver-child interaction in museums (e.g., Callanan et al., 2020; Medina and Sobel, 2020; Sobel et al., 2021), scores of 1 or 2 indicated that the interaction was entirely or mostly child-directed, scores of 3 indicated collaboration, and scores of 4 or 5 indicated that the interaction was mostly or entirely caregiver-directed. Each video was coded independently by two coders, who met with the second author after every 5 videos to reconcile any discrepancies. The average Cronbach’s alpha for the different pairs of coders was 0.71.

#### Juicy moments

Because we had no direct measure of children’s learning, we aimed to draw out moments of potential learning from children’s exploration videos. The first author and a team of four coders developed a coding scheme to capture such “juicy moments,” in which children were engaging with exhibits and/or with other individuals in such a way that indicated that they were learning something, changing their minds, or having a particularly important experience. For example, when looking at a fossilized fish, one participant said, “But really, fishes do not have bones. So that’s the only fish that looks like it has bones.” She was corrected by her father, who said, “No, that’s not true, fishes have bones,” to which she responded, “Oh!” Although these moments could be indications of engagement, this coding scheme is importantly distinct from our coding of children’s overall engagement in its focus on specific moments in children’s exploration and in its focus on indications of potential learning, beyond general excitement or enthusiasm. For this coding scheme, each participant was assigned to two coders who worked independently. They watched the GoPro footage that children had generated and noted the timestamp of each juicy moment, and they periodically reconciled their codes under supervision from the first author. Agreement on the final set was 99.7%.

#### Reflections on exploration

During the reflection interview, children were first asked why they approached that exhibit. This response was coded for whether children articulated a reason that was intrinsic (e.g., “I wanted to learn about the dinosaur,” “I wanted to try the walking”) or a reason that was either more descriptive or extrinsic (“Julia [sister] was over there,” “Dinosaurs are big”).

Children were then asked what they were doing and thinking about at the exhibit. Responses to this question were coded for whether they conveyed factual information about dinosaurs or another facet of the exhibit, beyond just an observation of what they had said or done (e.g., “That the holes that are on the tailbone were from teeth”).

A subset of the data (20 videos or 38% of the sample) were coded by two undergraduate research assistants, blind to the hypotheses of the study and children’s age or any other demographic information. Agreement for the coding of intrinsic vs. extrinsic reasons was 90.7%, Kappa = 0.81. Agreement for the coding of whether children provided factual information in their reflections was 92.3%, Kappa = 0.82. Disagreements were resolved through discussion. One of those coders then coded the remaining data.

#### “Did you learn something?”

The post-exploration interview asked children if they learned anything. Children who said “yes” were then asked what they learned. Responses to this question were coded as either referring to content (e.g., “dinosaurs can be really small”) or to a process or strategy for learning (e.g., “it was cool to read all those things I did not know”). They were then asked how they learned. These responses were coded as describing learning either with respect to behaviors (e.g., “I looked inside the skulls”) or with respect to mental states (e.g., “I thinked about how that’s how dinosaurs grow”).

Children who said “no” to the initial question of whether they had learned something were then asked what they were doing. Responses to this question were coded as either referring to behaviors (e.g., “just to look at prehistoric animals”) or to mental states (e.g., “thinking about stuff”). They were then asked if they could have been learning while doing that other activity, and they could say yes or no.

Two coders initially coded 20% of the sample to check for reliability on these two sets of codes. Agreement on this subset was 90%, Kappa = 0.86. One coder then coded the rest of the sample.

## Results

### Children’s experiences in the exhibition

Supplemental Table S1 provides descriptive information about each exhibit, including the total number of visitors and the average amount of time spent there.

Children made an average of 10 visits to exhibits during their exploration time (Range 2–27); these numbers include times when they returned to a previously visited exhibit. Children visited an average of 9 unique exhibits (Range 2–18). They made an average of 7 visits to static exhibits (Range 0–27) and 3 visits to interactive exhibits (Range 0–8).

In terms of time spent, children were actively visiting exhibits during their 10-minute exploration time (as opposed to transitioning between exhibits) for an average of 448 seconds (Range 102–808 seconds). They spent on average 215 seconds at static exhibits (Range 0–749 seconds, average proportion of total exploration time 46.4%) and 233 seconds at interactive exhibits (Range 0–619 seconds, average proportion of total exploration time 53.6%).

We identified an average of 0.73 juicy moments per exploration, with more of such moments occurring at the static exhibits (*M* = 0.55) than at the interactive exhibits (*M* = 0.18).

### Child engagement during exploration

One of our primary questions for this project was to investigate what factors would relate to child engagement in the exhibition. Table 1 shows the zero-order correlations among children’s engagement score and their age, as well as the relations among those variables and the time spent exploring and whether the exhibits encouraged children to have a juicy moment.

**Table 1:** Correlations among children’s engagement, age, time spent exploring, and juicy moments.

	Children’s engagement (scale of 1–5)	Time exploring static exhibits	Time exploring interactive exhibits	Number of juicy moments at static exhibits	Number of juicy moments at interactive exhibits
Children’s age	0.10	0.37*	−0.40*	−0.002	−0.31*
	*p* = 0.47	*p* = 0.007	*p* = 0.004	*p* = 0.99	*p* = 0.03
Children’s engagement		0.36*	−0.003	0.41*	0.09
		*p* = 0.008	*p* = 0.98	*p* = 0.003	*p* = 0.55

A * indicates a statistically significant result.

Our analyses first considered the extent to which children explored each exhibit and its relation to their engagement and to the nature of their interaction with their caregivers. There was no relation between the length of time children explored and their age, *r*(49) = 0.004, *p* = 0.98. However, older children spent more time at static exhibits, *r*(49) = 0.37, *p* = 0.007, and younger children spent more time at interactive exhibits, *r*(49) = −0.40, *p* = 0.004. Boys and girls did not differ in the overall amount of time children spent exploring, or in the amount of time they spent exploring either the static or interactive exhibits, all Mann Whitney Tests, |z| < 0.80, all *p*-values >0.42.

Children’s engagement with their exploration was rated 3.94 on average (Range 2–5). Boys (*M* = 3.93) and girls (*M* = 3.95) were no different in their overall level of engagement. Children’s engagement scores correlated positively with the total time children spent exploring, *r*(49) = 0.55, *p* < 0.001. That is, the more time children spent exploring, the more engaged they were judged to be. That correlation was also significant when controlling for age, *r*(48) = 0.55, *p* < 0.001. As can be seen in Table 1, the amount of time children spent at the static exhibits correlated with their engagement, and this relation held when age was controlled for, *r*(48) = 0.35, *p* = 0.01. However, the amount of time children spent at the interactive exhibits did not relate to their engagement. As can also be seen from Table 1, the juicy moments that happened at the static exhibits related to children’s engagement; this correlation was also significant controlling for age and the amount of time children spent at the static exhibits, *r*(46) = 0.41, *p* = 0.003.

We next considered the relation between children’s engagement and the extent to which caregivers guided their children through the exploration, as defined by the three categories of caregiver-child interaction style. Collaborative dyads (*n* = 15) spent more time exploring overall (Mean = 461.40  seconds, SD = 83.37) than caregiver-led dyads (*n* = 12, Mean = 449.66 seconds, SD = 75.25) and child-led dyads (*n* = 25, Mean = 438.20 seconds, SD = 147.71). Children in collaborative dyads were also rated as more engaged (Mean = 4.33, SD = 0.72) than children from caregiver-led (Mean = 3.75, SD = 0.45) or child-led (Mean = 3.80, SD = 0.96) dyads. Collaborative dyads also generated more juicy moments during the course of their exploration (*M* = 1.14) than either caregiver-directed (*M* = 0.58) or child-directed (*M* = 0.56) dyads. This was specifically the case for static exhibits, where collaborative dyads generated more juicy moments (*M* = 1.00) than the other two groups (*M* = 0.33 and *M* = 0.40, respectively). However, neither of these differences were statistically significant, Kruskal–Wallis *H*(2) = 2.66 and 4.25, *p* = 0.27 and 0.12.

To analyze these data together, we constructed a set of hierarchical regression models. The first model predicted children’s engagement score from their age, the time spent exploring the interactive exhibits, the number of juicy moments that occurred at the interactive exhibits, and the number of static exhibits and number of interactive exhibits that children visited. These latter two variables were included to control for the fact that there were more static exhibits in the exhibition than interactive ones. This model did not explain a statistically significant amount of variance, *R*^2^ = 0.20, *F*(5,44) = 2.25, *p* = 0.06. We then added caregiver-child interaction style to this model. This new model predicted a significant amount of additional variance, Δ*R*^2^ = 0.13, *F*(2,42) = 3.97, *p* = 0.03, with children in the collaborative group being more engaged than children in the child-directed group, *B* = 0.37, *p* = 0.009. We then added the time children spent only at the static exhibits, and this also predicted a significant amount of additional variance, Δ*R*^2^ = 0.12, *F*(1,41) = 9.08. Finally, we added to the model the number of juicy questions children generated at the static exhibits, which also predicted a significant amount of additional variance, Δ*R*^2^ = 0.06, *F*(1,40) = 4.42, *p* = 0.04. This final model was significant overall, *R*^2^ = 0.51, *F*(9,40) = 4.56 *p* < 0.001.

To summarize, this set of analyses examined what factors related to children’s engagement with their exploration of the exhibit. We found that the time children spent at static exhibits and the number of juicy moments at those exhibits were most predictive of their engagement: Children who spent more time and who generated more juicy moments with their families at those exhibits were rated as more engaged.

### Post-exploration reflections

Our next research question investigated how children talked about their exploration, particularly in terms of the motivation they had for their actions and the extent to which they understood the content of the exhibits. In general, children provided reflections on 2–9 exhibits (Mean = 5.00, SD = 1.67) in their post-exploration interviews.

We first considered how children described their decision to go to a particular exhibit during these reflections.^5^ Overall, children stated that their reason for visiting an exhibit was intrinsic to their interests on 44% of their reflections. We analyzed these data with a Generalized Estimating Equation with a robust correlation matrix, to control for the within-subject nature of the question, assuming a binomial response. Age, caregiver-child interaction style, whether the exhibit was static or interactive, the total time children spent exploring, and the order of the reflections were the independent variables. All main effects were considered as were interactions concerning the first four variables (because there is no hypothesized reason why interactions with order of reflection would be significant). Interactions were removed from the model if they were non-significant, and the resulting model was a better fit for the data, as indicated by lower QICC values. The final model considered all the main effects as well as the interaction between age and the total time children spent exploring. Exhibit type (static or interactive) was not a significant factor in this model, Wald χ^2^(1) = 0.48, *p* = 0.49; neither was order of reflection, Wald χ^2^(1) = 2.49, *p* = 0.12. Children’s age was a non-significant trend, Wald χ^2^(1) = 3.07, *p* = 0.06. The only significant differences were in the caregiver-child interaction styles, with children in child-led dyads showing higher levels of intrinsic motivation in their reflections (48%) than children in caregiver-led dyads (33%), *B* = 1.14, SE = 0.52, 95% CI [0.12, 2.16], Wald *χ*^2^(1) = 4.84, *p* = 0.03, and in the effect of total time spent exploring, *B* = −0.03, SE = 0.01, 95% CI [−0.05, −0.004], Wald χ^2^(1) = 5.16, *p* = 0.02, and the interaction between time spent exploring and age, *B* = 0.0001, SE = 0.0001, 95% CI [0.000003, 0.001], Wald *χ*^2^(1) = 4.94, *p* = 0.03. To investigate this interaction further, we performed a median split by age. For the younger half of the sample (children under 98.10 months of age, or approximately 8 years), children who said that they were intrinsically motivated to go to an exhibit explored longer overall (459  seconds vs. 442  seconds), while the older half of the sample showed the reverse pattern (440 seconds vs. 447 seconds). Neither of these differences, however, were significant, both *r_s_*-values <0.05, both *p*-values >0.64.

We next considered whether children generated factual information regarding the exhibits in their reflections, which they did on an average of 30% of their reflections. We used the same analytic strategy on these data, looking at age, exhibit type (static or interactive), caregiver-child interaction style, total time exploring, and order of reflection. The final model here found no significant effect of reflection order, Wald *χ*^2^(1) = 0.66, *p* = 0.42. Exhibit type was a significant predictor: Children generated factual information when they reflected on static exhibits 33% of the time, significantly more often than they did so when they reflected on interactive exhibits (24% of the time), *B* = 6.67, SE = 2.03, 95% CI [2.68, 10.65], Wald χ^2^(1) = 10.74, *p* = 0.001. There were also significant effects of age and total time spent exploring. The mean age of children who generated factual information in a reflection was 100.31 months, while the mean age of children who did not was 96.09 months, *B* = −0.15, SE = 0.06, 95% CI [−0.27, −0.03] Wald *χ*^2^(1) = 5.74, *p* = 0.02. The mean time spent exploring when children generated factual information was 473 seconds, compared with 438 seconds when they did not, *B* = −0.04, SE = 0.01, 95% CI [−0.06, −0.01] Wald χ^2^(1) = 9.14, *p* = 0.003. There were also three significant interactions, between the caregiver–child interaction style and whether the exhibit was static or interactive, Wald χ^2^(2) = 7.38, *p* = 0.03, between age and exhibit type, Wald χ^2^(1) = 8.13, *p* = 0.004, and between age and time spent exploring Wald χ^2^(1) = 7.07, *p* = 0.008.

To consider the interactions with age further, we first performed a median split on the data set by age and reran the GEE analysis, focusing only on the difference between the static and the interactive exhibits and the total time spent exploring. In the younger half of the sample, children showed a trend to be more likely to generate relevant factual information for static exhibits (29% of the time) than interactive exhibits (17% of the time), *B* = 0.61, SE = 0.36, 95% CI [−0.10, 1.33], Wald χ^2^(1) = 2.85, *p* = 0.09. Children in the older half of the sample were also numerically more likely to generate relevant factual information for static exhibits (37% of the time) than interactive exhibits (32% of the time), but this difference was not statistically significant, Wald χ^2^(1) = 0.24, *p* = 0.63. Similarly, when children in the younger half of the sample generated factual information in their reflections, they explored the exhibits overall for longer (512 seconds) compared with when they did not generate factual information in their reflections (431 seconds); this was a significant difference, *B* = −0.01, SE = 0.002, 95% CI [−0.01, −0.002], Wald *χ*^2^(1) = 7.10, *p* = 0.008. In the older half of the sample, there was no significant difference in time spent exploring when children generated factual information in their reflection (447 seconds) and when they did not (448 seconds), Wald *χ*^2^(1) = 0.01, *p* = 0.94.

Finally, we looked at the relation between caregiver–child interaction style and when children generated factual information. The main finding of interest here was that caregiver-directed children who were in the older half of the sample generated factual information in 71% of their reflections, compared with 20% for caregiver-directed children in the younger half of the sample. Child-directed dyads (35% vs. 28%) and collaborative dyads (29% vs. 24%) did not show this difference. Of importance, however, is that the majority of caregiver-directed children were in the younger half of the sample, and thus the 71% value indicates the reflections of only two children.

To summarize the findings in this section, children who were able to direct their own exploration were more likely to report internally motivated reasons for their exploration of particular exhibits. This suggests that the children we tested might have been better able to reflect on their motivations for their exploration when caregivers were less involved in setting goals for the interaction. Further, as children got older, they were more likely to be able to talk about the content of the exhibits, particularly when the exhibits they visited were static, suggesting that older children in the sample were more likely to be learning from those exhibits.

### Children’s reports on their own learning

Our final question looked at whether children reported that they learned something during their exploration, and what factors motivated reporting that they learned. Overall, 80% of the children stated that they learned something during their exploration of the exhibits. We examined whether there were significant correlations between children stating that they learned something during their exploration and the time spent exploring the static and interactive exhibits, the number of juicy moments at each type of exhibit, their overall level of engagement, and their caregiver-child interaction style. We only found two significant effects. First, there was a significant correlation between children stating that they learned something from their exploration and the level of engagement they showed during their exploration, *r*(49) = 0.32, *p* = 0.02. Second, there was a significant correlation between children stating that they had learned something and the number of juicy moments they experienced during their exploration of the static exhibits, *r*(50) = 0.30, *p* = 0.03. No other correlation was significant.

To examine these variables’ independent effects, we constructed a binary logistic regression. While the overall model was significant, χ^2^(2) = 8.91, *p* = 0.01, only children’s engagement predicted variance in children stating that they learned something, and only at a marginally significant level, *B* = 0.90, SE = 0.52, Wald χ^2^(1) = 3.02, *p* = 0.08, Odds Ratio = 2.47. Thus, children’s reports about their learning seemed most influenced by their engagement with their exploration, and not any specific facet of the exploration itself.

## Discussion

In this study, we recorded children’s naturalistic exploration of a dinosaur exhibit in a natural history museum from a first-person perspective. We also interviewed these children following their exploration to gain further insight into how they viewed their experiences in the museum and how they thought about learning in general, using the videos they had generated as visual reminders. With this rich set of data, we can illuminate children’s experiences in informal learning environments and explicate the role of different influences on children’s exploratory behavior and their views of their own learning. In this way, this project can help us to gain a better understanding of how the exploration processes that we observe in the lab can unfold in real-world informal learning settings like museums.

The current analyses specifically aimed to investigate aspects of caregiver-child interactions in the exhibition and how these interactions related to children’s engagement and to their reflections on their experiences in the exhibition. We found that children were more engaged with the exhibits when they interacted collaboratively with their caregivers, as compared to when they or their caregivers were leading the interactions. This aligns with results of earlier studies on caregiver–child interactions in museum settings, in which collaborative interactions led to the most engagement (e.g., Medina and Sobel, 2020; Leonard et al., 2021; Sobel et al., 2021; Sobel and Stricker, 2022).

We also found that children were more engaged the more they visited more static exhibits, which presented fossils and bones with explanatory plaques, than when they visited more interactive exhibits, at which they could pretend to dig for fossils or run on a treadmill to make a dinosaur skeleton move. Although older children spent more time at the static exhibits and less time at the interactive ones, the relation between children’s level of engagement and time spent at the static exhibits held when controlling for children’s age. Children were also more likely to generate particularly rich observations or interactions, which we called juicy moments, at static exhibits than at interactive exhibits. Exploration at the static exhibits also led to children reporting on more factual information, beyond simple descriptions of their actions at the exhibit, in their post-exploration interviews – a clue that they may have learned more from these exhibits.

These results are perhaps surprising from the point of view of museum design, because interactive exhibits provide more opportunity for children to choose their own actions and potentially to learn more or engage more deeply (see Falk et al., 2002; Falk and Dierking, 2018). Indeed, children in this sample tended to spend more time at the interactive exhibits, both overall and proportionally, which provides at least one indication that such exhibits were interesting to them.

To explain this pattern, we believe that it is productive to put this result into context with the relation between child engagement and caregiver-child interaction style. We found that children were more engaged when they interacted collaboratively with their caregivers, and static exhibits provide more opportunities for this kind of engagement. Caregivers tended to remain more hands-off when children were digging for fossils or playing in a green-screen room that allowed them to pretend to interact with computer-generated dinosaurs. Potentially, these interactive exhibits did not allow for the kind of collaborative interactions that led to higher engagement. Further, older children in the sample spent more time at the static exhibits and less time at the interactive ones. One possible reason for this could be that older children might be seeking out more collaborative caregiver–child interactive opportunities, although future work should aim to explore this relation in more detail. Understanding more about what leads to deep and genuine engagement at museum exhibits can benefit both museum design and our understanding of how children may learn in these naturalistic settings.

Finally, with respect to children’s views of their own learning, children were more likely to say that they learned something during their museum exploration when they were coded as being more engaged by their exploration. We again saw an advantage for the time children spent at the static exhibits for this relation. Older children in particular tended to generate more factual information in their post-exploration reflections (information above and beyond descriptive information about what they had done or seen) when reflecting on their experience at a static rather than an interactive exhibit.

Although all of these findings require further investigation, they have the potential to translate into recommendations for museum practices. One of the primary recommendations from the current study would be to encourage more collaboration between children and caregivers in museum settings, perhaps through signage or guidance from staff. A second recommendation would be to think carefully about an exhibition’s balance between static and interactive exhibits (see Dancstep et al., 2015): Children (especially younger children) enjoy interactive elements, but static exhibits seem to have an important role to play in children’s engagement. Both greater engagement and longer dwell times at static exhibits related to children’s generation of juicy moments; insofar as museums are aiming to encourage such moments, exhibit design could take these relations into account. Finally, the older children in the dataset were potentially more able to draw out educational messages from interactive exhibits than younger children, suggesting that exhibit design and messaging should be sensitive to the ways in which interactive exhibits may be interpreted differently by visitors of different ages.

### Limitations and future directions

One of the main strengths of this project is in the rich, qualitative data that we have collected, particularly the first-person videos recorded by the children in this study. Because these data were collected within a naturalistic setting, with no direction from researchers about how to engage with the exhibition, they offer a unique view into children’s genuine interactions in a museum environment. However, this choice of method also has several weaknesses, most notably in its lack of control. Children were allowed to explore the space in any way that they wished, meaning that not all of our participants saw all exhibits, and our participants explored these exhibits in different orders. Additionally, we put no restrictions on the kinds of interactions that children could have, meaning that some interacted with museum staff while others did not. Our dataset also includes several different types of family groupings, including multiple adults with a single child, multiple children with a single adult, and many others. While this tradeoff between naturalism and control allows us to be confident that our findings reflect a wide range of responses to our target exhibition, it does not allow us to go beyond the correlational results reported here. Future work should build on the current findings to investigate more fully how children and adults interact at static versus interactive exhibits, for example, which would allow us to strengthen the current conclusions about the value of static exhibits for enhancing children’s engagement.

Another important limitation is that we only investigated children between the ages of 6 and 10 years old. Expanding our age range could allow us to add nuance to the current findings, since interactivity in exhibits is often geared toward younger children as a way of encouraging their engagement with museum content (particularly STEM content). Further, the older children in our sample might come to the museum with different exploratory goals than younger children. Along these lines, in this study, we were not able to consider what goals children had for their exploration of the exhibition prior to letting them explore. We did collect relevant information from the children’s caregivers in our demographic questionnaire, but these did not relate to children’s exploration or the caregiver-child interaction style (see the Supplementary materials section for details). Future research, however, could potentially interview children prior to their exploration as to what goals they have for their visit to the museum, and then see how their exploration is shaped by those goals. Future research could also focus more directly on the content of the exhibit, measuring children’s knowledge about or interest in dinosaurs before and after their visit to the exhibit. This could help to clarify the extent to which the pedagogical goals of the exhibit are being met and what kinds of exploratory behaviors are most strongly associated with that kind of direct content learning.

Finally, our approach to analyzing these data has been to transform it into quantitative codes that align with previous literature in this area (e.g., Callanan et al., 2020), but we fully acknowledge that this does not capture the depth of what is happening in these videos. By making them public to the extent that we can (see footnote 3), we hope that other researchers will be able to apply their own analysis strategy to children’s exploration in these videos and to use them as a resource to explore a wide range of other questions. This can enable the field to understand in more detail how children engage with and learn within informal learning environments.

## Data availability statement

The datasets presented in this study can be found in online repositories. The names of the repository/repositories and accession number(s) can be found below: https://osf.io/8xghm/.

## Ethics statement

The studies involving human participants were reviewed and approved by Institutional Review Board of Villanova University. Written informed consent to participate in this study was provided by the participants’ legal guardian/next of kin. Written informed consent was obtained from the individual(s), and minor(s)’ legal guardian/next of kin, for the publication of any potentially identifiable images or data included in this article.

## Author contributions

DW and DS jointly conceptualized the idea for this study and secured funding. DW was responsible for data collection, overseeing data coding, and writing the first draft of the manuscript. LD implemented two of the main coding schemes for the data, supervised by DW. DS conducted the analyses and implemented two other coding schemes. All authors have read, edited, and approved the submitted version of the manuscript.

## Funding

This research was supported by the National Science Foundation (1661068 and 1917639 to DS, and 1929935 to DW). This work received additional funding from Villanova University’s Falvey Library Scholarship Open Access Reserve (SOAR) Fund.

## Conflict of interest

The authors declare that the research was conducted in the absence of any commercial or financial relationships that could be construed as a potential conflict of interest.

## Publisher’s note

All claims expressed in this article are solely those of the authors and do not necessarily represent those of their affiliated organizations, or those of the publisher, the editors and the reviewers. Any product that may be evaluated in this article, or claim that may be made by its manufacturer, is not guaranteed or endorsed by the publisher.

## Footnotes

^1^One parent did not provide their child’s exact birthdate but did confirm that the child was in our age range. This child’s data was not included in any analysis reported below concerning age, hence the different degrees of freedom for those analyses.

^2^ https://osf.io/8xghm/

^3^ http://doi.org/10.17910/b7.854

^4^ https://osf.io/8xghm/

^5^One child chose not to stay for the post-exploration interviews, so these analyses are conducted on the remaining 51 participants.
