# Supplementary material for: Dinos and GoPros: Children’s exploratory behaviors in a museum and their reflections on their learning
Source: Front Psychol. 2023 Feb 13;14:1110612. doi: 10.3389/fpsyg.2023.1110612 (PMC9968754; doi:10.3389/fpsyg.2023.1110612)
Supplement: Supplementary file 1 [file Data_Sheet_1.pdf]

Supplementary Materials for "Dinos and GoPros: Children's exploratory behaviors in a museum and their metacognitive understanding of learning"

Description of Exhibition

The Dinosaur Hall exhibition at the Academy of Natural Sciences stretches across two floors and is takes up approximately 10,000 square feet. It contains more than 30 species of dinosaurs and other Mesozoic reptiles, about half of which are full skeletal mounts. Descriptions of the different exhibits within this exhibition can be found in Table S1. See OSF (https://osf.io/8xghm/) for a map of the exhibition and thumbnail photographs of each exhibit.

Table S1. Description of exhibits within Dinosaur Hall

Exhibit	Exhibit Type	Floor	Total number of visitors to this exhibit	Average number of seconds spent at this exhibit	Average percentage of time spent at this exhibit	Total number of juicy moments at this exhibit
1. Discovering Dinos: An introduction to the space that prompted visitors to ask questions about how we know what we know about dinosaurs	Static	1	11	59.64	11.84	1
2. Family: A grouping of dinosaurs depicting an adult dinosaur protecting several young from a predator	Static	1	28	39.11	8.61	1
3. Footprint: A large cast of a dinosaur footprint that visitors could sit in	Static	1	13	16.23	3.37	0
4. Avaceratops: An Avaceratops skeleton	Static	1	17	29.65	6.36	1

5. Fossil Wall: An outline of a dinosaur on the wall with a few bones mounted within the outline, showing what paleontologists originally found	Static	1	11	25.73	6.10	1
6. Display Cases: Two glass display cases with artifacts (e.g., medal, microscope) associated with a famous paleontologist	Static	1	17	48.41	10.53	3
8. Torosaurus: A Torosaurus skull	Static	1	13	26.00	5.65	2
9. Fossil Lab: Paleontologists work on fossils that were brought back from digs on one side of a plexiglass barrier and visitors can observe them working and ask questions	Interactive	1	20	86.00	18.34	1
10. Green Screen Room: Visitors can walk inside and pretend to interact with computer-generated dinosaurs on a screen	Interactive	1	28	69.39	17.50	1
11. Green Screen Screen: Display of the output from the green screen room, showing visitors interacting with dinosaurs	Interactive	1	19	34.26	7.43	0
12. Partial Skull: A rock with a dinosaur skull embedded in it	Static	1	6	17.83	4.73	0
13. Turtles: Several skeletons of ancient relatives of turtles, suspended from the ceiling as if swimming	Static	1	20	39.70	8.12	1
14. Marine Reptiles: Skeletons of extinct	Static	1	26	51.23	12.04	4

marine reptiles, suspended from the ceiling as if swimming						
15. Dolphin Case: A skeleton of an aquatic dinosaur embedded in rock	Static	1	12	25.42	5.36	1
16. Marine Reptile Wall: Paintings of what the marine reptiles from the main fossil display may have looked like	Static	1	4	16.75	3.49	0
17. Legs: A series of femurs from a chicken, and human, and several dinosaurs so that visitors can compare sizes and shapes	Static	1	5	13.00	3.06	0
18. Skulls: Three skulls from related dinosaurs mounted in a group to enable comparisons	Static	1	12	41.5	8.35	1
19. T-Rex: A T-Rex skeleton	Static	1	21	32.20	7.09	2
20. Triceratops: A Triceratops skeleton	Static	1	19	32.47	8.23	4
21. Hadrosaur: A Hadrosaur skeleton	Static	1	21	27.62	7.73	2
22. T-Rex Femur: A cast of a partial femur from a T-Rex	Static	1	2	29.00	5.52	1
23. Big Skulls: A skull of a T-Rex and of a Triceratops that visitors can climb inside	Interactive	2	22	44.50	9.92	3
24. Animal Comparisons: Several displays comparing modern animals to dinosaurs (e.g., a dinosaur skull accompanied by text discussing how it's similar to an armadillo)	Static	2	12	37.08	8.47	0

7. Triceratops Case: A small model of a triceratops in a glass case	Static	2	5	12.60	2.72	0
25. Eggs: A collection of fossilized dinosaur eggs in a glass case	Static	2	10	22.00	5.66	2
26. Stegosaurus: A model of a Stegosaurus mounted on the wall with the skin and flesh removed so that the internal organs are visible	Static	2	13	19.08	4.54	0
27. Walk like a Croc: Two strips of footprints on the floor challenging visitors to follow along in order to walk like a crocodile (on all fours, with the limbs splayed out) and like a dinosaur (upright, with the legs underneath the body)	Interactive	2	14	31.50	9.90	0
28. Chicken: Comparisons of a modern chicken skeleton to those of two small dinosaurs	Static	2	6	12.67	3.41	0
29. Prey and Predator: A series of fossils in cases showing predator-prey interactions (e.g., a bone showing teeth marks)	Static	2	7	26.86	8.40	0
30. Treadmill: The treadmill was connected to an articulated dinosaur skeleton in a case next to the treadmill, so that walking on the treadmill made the dinosaur appear to run	Interactive	2	14	46.71	14.79	0

31. Bird: A skeleton of a flying dinosaur embedded in rock	Static	2	1	10.00	3.33	0
32. Big Dig: Casts of dinosaur bones were embedded in the floor and covered with pieces of shredded cork, so that visitors could pretend to dig for dinosaurs by brushing away the cork with their hands or with brushes provided in the exhibit	Interactive	2	28	218.68	50.56	4
33. Paleontology Wall: A display of tools that paleontologists use when digging for dinosaurs	Static	2	4	22.50	5.66	1
34. Pteranodon: A skeleton of a Pteranodon, suspended from the ceiling as if flying	Static	2	6	29.67	6.07	0
35. Special Exhibit (Chickens): Live chickens were brought into the exhibit during one the days when we were testing to provide a comparison with the structure of dinosaur skeletons	Interactive	2	1	138.00	31.01	0

Demographics of the Sample

Child Gender

The final sample consisted of 52 children. There were 30 boys and 22 girls. When considering the variables analyzed in the manuscript, none were significantly different based on gender. This included

- Children's engagement (Boys, 3.93; Girls, 3.95 out of a possible 5), Mann-Whitney U = 323.50, z = -0.13, p = .90.
- Total time spent exploring (Boys, 457 seconds; Girls 424 seconds), Mann-Whitney U = 286.00, z = -0.82, p = .42.
  - Time spent exploring static exhibits (Boys, 192 seconds; Girls 213 seconds), Mann-Whitney U = 303.00, z = -0.50, p = .62.
  - Time spent exploring interactive exhibits (Boys, 264 seconds; Girls, 211 seconds), Mann-Whitney U = 274.00, z = -1.04, p = .30.
- Number of juicy moments during exploration (Boys, 0.66; Girls, 0.81), Mann-Whitney U = 284.00, z - -0.65, p = .52.
- Whether children said that they learned during the reflection interview (Boys, 79%; Girls 82%), $\chi^2(1) = 0.05$, p = .82
- Caregiver-child interaction style (Boys: 7 Parent Directed, 6 Jointly Directed, 17 Child Directed; Girls: 5 Parent Directed, 9 Jointly Directed, 8 Child Directed), $\chi^2(2) = 3.01$, p = .22.

Caregiver Gender

Of these 52 children, 28 participated with a female caregiver and 24 participated with a male caregiver. When considering the variables analyzed in the manuscript, none were significantly different based on the caregiver's gender. This included

- Children's engagement (Male, 3.92; Female, 3.96 out of a possible 5), Mann-Whitney U = 326.00, z = -0.20, p = .85.
- Total time spent exploring (Male, 433 seconds; Female 453 seconds), Mann-Whitney U = 252.00, z = -1.54, p = .12.

- Time spent exploring static exhibits (Male, 222 seconds; Female 183 seconds), Mann-Whitney U = 300.50, z = -0.65, p = .52.
- Time spent exploring interactive exhibits (Male, 210 seconds; Female, 268 seconds), Mann-Whitney U = 251.00, z = -1.56, p = .12

- Number of juicy moments during exploration (Male, 0.65; Female, 0.79), Mann-Whitney U = 298.00, z - -0.50, p = .62.
- Whether children said that they learned during the reflection interview (Male, 78%; Female 82%), $\chi^2(1) = 0.12$, p = .73
- Caregiver-child interaction style (Male: 3 Parent Directed, 7 Jointly Directed, 14 Child Directed; Female: 9 Parent Directed, 8 Jointly Directed, 11 Child Directed), $\chi^2(2) = 3.14$, p = .21.

Education Level and Household Income

Responses to our questionnaire about education level was divided into four categories: (1) Attended high school, but no further education (6 respondents), (2) Associates Degree/Attended Trade School/Some College (13 respondents), (3) Bachelor's Degree or equivalent (18 respondents), (4) Obtained Graduate Degree (15 respondents).

Household income was divided as follows: Less than 30K (1 respondent), 31-50K (6), 51-70K (3), 71-90K (9), 91-120K (11), 120-150K (7), above 150K (11). Four families did not respond to this question.

Neither of these factors significantly correlated with any of the variables under consideration, all $|r_s$-values$| < .22$, all p-values > .13. Caregivers did not differ in either household income or education level across the three types of caregiver-child interaction style,

both Kruskal-Wallis H(2)-values < 2.58, both p-values > .27. Unsurprisingly, parental education level and household income significantly correlated with one another, $r_s(46) = .57$, $p < .001$.

Attitudes Towards Science Questionnaire

While participants engaged in the post-exploration interview, parents or guardians filled out a questionnaire to provide more information about their family. The questionnaire also assessed the caregiver's attitudes about science (Szetcher & Carey, 2009) by asking for their level of agreement with 15 statements (e.g., "The world would be better off if people thought more like scientists"; "Science makes me feel like I am lost in a jumble of numbers and words", reverse-coded). Caregivers indicated their agreement with these statements on a scale of 1 (mostly disagree) to 7 (mostly agree). The full questionnaire can be found on OSF (https://osf.io/8xghm/).

Forty-nine caregivers responded to this questionnaire. Their average score was 5.20 out of a possible 7, where higher scores indicate greater interest in and valuing of science. Scores ranged from 3.80 to 6.40. These scores did not significant correlate with any of the variables under consideration, all $|r\text{-values}| < .22$, all p-values > .13. Interestingly, scores on this questionnaire did relate to caregiver-child interaction styles, with caregivers in caregiver-directed dyads (n=11) scoring lower (Mean=4.88; SD=0.77) than caregivers in collaborative dyads (n=14; Mean=5.57, SD = 0.58) and lower than caregivers in child-directed dyads (n=24; Mean=5.12, SD = 0.52), One-Way ANOVA, $F(2, 46) = 4.48$, $p = .02$. This relation held when controlling for children's age $\Delta R^2 = .16$, $F(2, 45) = 4.39$, $p = .02$.

While this finding is interesting, it is important to note that several other studies that have examined caregiver-child interaction using similar coding schemes have also administered this same Attitudes towards Science questionnaire, and have not found any relation between the way

parents and children interact and scores on this measure (Callanan et al., 2020; Sobel et al., 2021). Care, thus, must be taking in interpreting this finding, and reproduction is necessary before conclusions are made.

Liking Dinosaurs

At the end of the interview, we asked parents, “Compared to other children, I would describe my child as liking dinosaurs,” and provided the answer options “not at all,” “a little”, “somewhat”, and “a lot”. Of the 48 parents who responded to this question, 1 selected “not at all” (2%), 4 selected “a little” (8%), 16 selected “somewhat” (33%), and 27 selected “a lot” (56%).

Boys were significantly more likely than girls to be rated as liking dinosaurs a lot, $\chi^2(3) = 12.78$, $p = .005$. However, there was no relation between this variable and the time children spent exploring (overall, at static exhibits, or at interactive exhibits; all F-values < 1.00, all p-values > .37), child engagement ($F(2) = 1.31$, $p = .28$), or caregiver-child interaction style ($\chi^2(6) = 9.64$, $p = .14$).

“What is Learning” Interview

At the end of the post-exploration interview, participants responded to two sets of questions designed to probe their understanding of learning in general (based on Sobel & Letourneau, 2015). First, they were first asked “what is learning?” and “how do you learn?”, and then, after providing a response, “are there other ways to learn?” One participant did not receive these questions due to experimenter error.

Children’s responses to these open-ended questions about learning were coded into 4 categories, following Sobel & Letourneau (2015). *Strategy* responses referred to ways that

learning can happen (e.g., "you get to know something new"). *Source* responses referred to people or items that could provide information to learn (e.g., "I learn from reading books and I also learn from looking on the PC"). *Content* responses referred to pieces of information that could be learned (e.g., "math, dinosaurs, reading"). *Identity* responses referred to learning as learning or were otherwise redundant (e.g., "something that helps you learn more"). We also asked children how they learn and if there were other ways to learn, these responses did not vary meaningfully among participants and will not be considered further.

Thirty-eight children provided strategy responses. Seven provided source responses. One child made an identity response and one made a content response. Four said, "I don't know" or provided no response of substance. Sobel and Letourneau (2015) found in their sample of 4-10-year-olds that children's appeals to sources and strategies in their definitions of learning (which they coded together as "process") increased with age, with 6-7-year-olds making many more such definitions than 4-5-year-olds. In our sample of 6-9-year-olds, there was not a significant relation between generating definitions of this type and children's age $r_s(48) = .16$, $p = .25$. However, overall, children were making such a response 88% of the time. This code was also unrelated to any other aspect of the analyses, including whether children stated that they learned during their reflections, $r_s(48) = .13$, $p = .38$.

Following this set of open-ended questions about learning, participants were shown a picture of a novel toy (taken from Bonawitz et al., 2011). They were told that three other friends found the toy but didn't know what to do with it. One of the friends said that they're going to ask their grown up; the second said that they're going to watch someone else play with it; and the third said that they're going to play with it themselves (order randomized for each participant). Participants were asked which they would do to learn about the new toy: ask a grown-up, watch

someone else, or play with it themselves. They were they asked to justify this response. Regardless of which option they chose, they were then asked whether it was possible to learn in each of these three ways[1].

Eleven children said that they would ask their grown up, 12 said they would watch another play with it, and 27 said that they would play with it themselves. The frequency with which children generated a process-based definition of learning on the first set of questions did not differ among children who made these three responses, Kruskal-Wallis H(2) = 3.19, p = .20. However, not all children justified their answers in a relevant manner: Only 9 children who said they would ask a grown up, 8 who said they would watch another, and 18 who said they would play with it themselves did so. When these 35 children were examined, there was a difference in the way they responded to the first question, with all of the children who stated that they would watch another or play with it themselves generating a process-based definition of learning, while only 66% of the children who said they would ask their grown up doing so, Kruskal-Wallis H(2) = 9.21, p = .01. While this findings suggests that children with more of an understanding of how they might go about learning recognize that learning itself is a process of acquiring knowledge, the small sample size here calls for further investigation and reproduction of this finding.

[1] Due to experimental error, some children were not asked these questions. However, children never said "no" in response to these questions, so they were not analyzed further.
